# General Education as Preparatory to Medical Studies

**Published:** 1881-07

**Authors:** 


### Editorial.

### GENERAL EDUCATION AS PREPARATORY TO MEDICAL STUDIES.

The following brief paper was prepared for presentation to the meeting of the American Medical College Association, in Richmond, last May. But as a quorum of delegates was obtained only once, and then only long enough to hear the report of the Secretary and elect officers for the ensuing year, no other business was transacted, and, of course, no papers presented for consideration. The subject of preliminary education, however, is of sufficient importance to justify the insertion of what we had written in these pages, though in form addressed to the members of the College Association. It is as follows :

The necessity for a reasonable amount of mental development and discipline, and the advantages to be derived from a fair knowledge of the common branches of education, including the mathematics, physics, and natural sciences, before entering upon the study of medicine, are too obvious to require either argument or illustration.

Yet it is well known to all the members of this Association that a large per cent. of those who enter upon the study of medicine in this country have neither the mental discipline nor the knowledge just alluded to ; and only a small number of the colleges enforce any rule on the subject. Of all the defects in our system of educating men for the practice of medicine and surgery, none are greater or productive of more injury to the community and to the profession itself, than this want of preparatory knowledge and discipline on the part of those who enter it. It narrows the field of mental vision ; limits the materials for comparison and criticism ; precludes the possibility of apprehending the philosophy or reason for many things, and consequently compels the acceptance of the opinions of teachers and writers without question; and embarrasses every step of intercourse either with professional brethren or with the community. These embarrassments and limitations to the progress and usefulness of individuals necessarily involve corresponding limitations to the usefulness and honor of the profession as a whole. Improvement here at the threshold of the profession, is naturally the first and most necessary step towards the accomplishment of needed improvements in all other respects.

Admitting the correctness of the foregoing statements, two questions of paramount importance demand the serious attention of this Association. First, What should constitute the minimum amount of education required, to qualify a person to enter upon the study of medicine? Second, When, and by whom, should the amount be ascertained and the standard enforced?

In considering the first of these questions it must be borne in mind that true education embraces both mental discipline and the acquisition of knowledge. The first imparts that capacity for thought, analysis, and deduction so necessary for successfully studying the various departments of medical science and art; and the second furnishes the implements or materials for efficient mental action. To acquire the amount of mental discipline here indicated, certainly requires direct scholastic training sufficient to acquire a good practical knowledge of the common English branches, with the addition of algebra, mental philosophy, physics, physical geography, and botany; while the knowledge embraced in these several branches of study may be regarded as an essential prerequisite to a proper study and understanding of the several branches of medical science. Taking the amount of education here indicated as the minimum standard preparatory to entering upon the study of medicine, I come to the second question, namely: When, and by whom should this standard be enforced?

All must agree that the time when the standard of preliminary education should be enforced is when the student proposes to enter upon his professional studies. First, because the mental discipline and knowledge resulting from such prior education are necessary to qualify him to prosecute his medical studies with facility and success. And second, because, to permit him to spend three years of time and a considerable sum of money in pursuing professional studies and then reject him for lack of acquirements which he should have had before he began, would be so manifestly unjust as to need no comment. If the standard is to be enforced when the student proposes to enter upon his professional studies, without appealing to legislative bodies, there are only three parties on whom the duty of such enforcement can be devolved, namely: the preceptor with whom the student is supposed to commence his studies—boards of censors or committees appointed by medical societies—and the medical college in which he proposes to receive instruction.

It requires but little reflection or a limited range of practical observation to show, that the relations of preceptor and pupil in medicine, at the present time, are so far nominal or fictitious, and withal so variable in the character and attainments of the parties concerned, that any attempt to obtain the enforcement of a standard of preliminary education by them would be entirely nugatory. An attempt to accomplish something in this direction has been made by some of the State and local medical societies. But the simple fact that the action of such societies, is binding on no one except their own members, and the further fact that one-third, if not one-half of all the practitioners in the United States are not members of any medical society, would render these society organizations entirely incompetent to enforce any general standard of education.

So far as influencing their own members is concerned they may accomplish much, and all their efforts in this direction should be encouraged.

It becomes evident, from the foregoing considerations, that the medical colleges are the only organizations belonging to the profession on which the duty of exacting a proper standard of preliminary education can be devolved with a prospect of success. We have fully arrived at that period of progress when those entering the profession so generally resort to the medical schools for the chief part of their instruction, that whatever conditions are exacted for the admission to these institutions will effectually reach the whole body of medical students. Hence, if a certain amount of education was agreed upon as the minimum that would be required for admission to registration and attendance in all our medical colleges it would effectually accomplish the object desired. It is only necessary that the faculty and trustees of each college should require all applicants for registration to furnish written evidence either in the form of diplomas or certificates from respectable colleges, academies or high schools that they have a good knowledge of all branches previously mentioned in this paper, or demonstrate their possession of the same by sustaining a satisfactory personal examination before a standing committee of the faculty, elected for that purpose. If this important measure could be adopted and carried into execution simultaneously by all the colleges, or even by all the members of this Association, it would neither alter their relative positions in relation to each other, nor so far diminish the aggregate number of students as to seriously embarrass any one of them; and yet it would cause a rapid improvement in the educational status of the great body of American medical students. This is clearly proved by the experience of the few colleges in which a fair standard of preliminary education has actually been demanded as a condition for admission for a sufficient number of years to test its practical working. In 1875, the faculty and trustees of the Chicago Medical College, which is also the medical department of the Northwestern University, adopted the standard of preliminary education already mentioned in this paper, except that botany was not included, and appointed a standing committee composed of three members of the faculty, to personally examine all applicants for admission to any of the classes in the college, who did not furnish the written evidences of qualification already specified. The same committee has been continued until the present, and has annually performed the duties assigned to it with fidelity. During the first two years a little more than twenty per cent. of all the applications for admission were either withdrawn rather than risk the results of an examination or were rejected by the committee. This ratio has steadily decreased, and the last year only ten per cent. of the whole number of applications were either withdrawn or rejected. This decrease does not result from any abatement of the preliminary requirements but from the uniformly better qualifications of the applicants, as illustrated by the fact that of the forty-six who graduated in 1875 only ten per cent. were graduates of literary colleges, while of the forty-six who graduated at the last public commencement, March 27, 1881, nearly thirty per cent. were graduates of literary colleges of good reputation, and ninety per cent. of the remainder had received a good academic training. Precisely similar results have followed the institution of admission examinations in the medical school of Harvard University.

President Eliot, in his last annual report, page 33, says, in reference to the medical school as follows: "The examination for admission was first held in 1877; and its good effects were so soon manifested that the faculty were ready, after only three years experience, to add to the requisitions * * * In this university, until the reformation of the school in 1870–71, the medical students were noticeably inferior in bearing, manners and discipline to the students of other departments; they are now *indistinguishable* from other students. A corresponding change in the medical profession at large would be effected in twenty years, if all the important medical schools of the country should institute a reasonable examination for admission." These statements of the President are fully sustained by the facts given in the annual report of the Dean of the medical school for the same years.

The simple truth is, that just so soon as the medical schools of this country establish any given standard of education to be required for *admission* to such schools, all persons intending to enter upon the study of medicine will make the required preparation without the least hesitation. But so long as the doors of the great majority of our medical colleges are open to all who come without the least regard to their general educational attainments, just so long will the evils and embarrassments that now mar the reputation and usefulness of the profession remain without essential improvement.

As stated, however, at the commencement of this paper, the necessity for the possession of a fair degree of mental discipline, habits of study, and actual knowledge of all those branches usually included in a good academic course of education, as a preparation for entering upon a field of study so extensive and intricate as that of medicine, is too apparent to justify further comment. And I confess to a feeling of disappointment, that, in the framing of the articles of confederation for this Association, one of its first provisions was not an *article* prescribing a matriculation examination of fair character to be instituted by each college as a condition of membership in the Association.

To remedy this great defect, I now propose as an amendment to Article II, of the articles of confederation a fourth section as follows:

" *Section* 4. Each college member shall require of all applicants for admission or registration as medical students, adequate written or documentary proof that said applicants have a thorough knowledge of the English branches including algebra, botany, physics, physical geography, and mental philosophy, or shall sustain a satisfactory examination on the same topics by a competent committee appointed for that purpose. N. S. D.

---

THE WARREN TRIENNIAL PRIZE.—The Warren Prize Committee offer a premium of $400 for the best Dissertation worthy of a prize, upon the following subject:—

" CHRONIC BRIGHT'S DISEASE (parenchymatous and interstitial nephritis). The Nature and Mutual Relations of the Derangements in the Circulatory and Secretory Organs."

Dissertations should be forwarded to the resident Physician, Massachusetts General Hospital, Boston, on or before February 1, 1883. J. COLLINS WARREN,

*Secretary Physicians & Surgeons, Mass. Gen'l Hospital.*

